# How to Win the Nobel Prize?

**DOI:** 10.1371/journal.pbio.0040333

**Published:** 2006-10-17

**Authors:** Peter Agre

## Abstract

One Nobel laureate reviews another's account of "The Beginner's Guide to Winning the Nobel Prize. A Life in Science."

Based on the title—*The Beginner's Guide to Winning the Nobel Prize*—one might expect Peter Doherty's new book to be a handbook targeted to the emerging group of anxious parents whose expectations for their youngsters' achievements have reached the stratospheric level. In truth, this is a delightful history and series of essays by an affable Australian veterinarian—sort of a James Herriott of science—who shared the 1996 Nobel Prize in medicine. I enjoyed sitting down with this book as much as I enjoy slipping on a comfortable old pair of shoes and sitting in our garden on a Saturday evening with a glass of wine. Doherty's book will make an interesting and entertaining read for scientists and will provide a delightful experience for the nonscientists, who may be impressed to learn how decent and truly normal an internationally renowned scientist can be.

Autobiographies have been published previously by several Nobel laureates. Although this volume is in some ways similar, it also bears many unique features. For example, Doherty's writings are much less gossipy than James Watson's infamous *The Double Helix* [1], and they are much more mainstream than Kary Mullis's gonzo-science as told in *Dancing Naked in the Mind Field* [2]. Doherty's book does not contain the heartbreak of a Jewish child in 1930s Vienna as revealed in Eric Kandel's *In Search of Memory* [3]; the volume is probably closer to J. Michael Bishop's *How to Win the Nobel Prize: An Unexpected Life in Science* [4], in which the amusing and whimsical side of science is presented.

Peter Doherty can never be accused of egotistical self promotion. As he reveals, science journalist Gina Kolata, upon learning of the 1996 Nobel announcements, considered Rolf Zinkernagel (Doherty's co-winner) normal enough, although his nickname “Hyper-Zink” may have been a clue that was missed. However, the same journalist described Doherty as “Eeyore-like,” in reference to A.A. Milne's depressive donkey. Whether this is accurate is unknown to me, but the image is unforgettable.

The description of the three years that Doherty and Zinkernagel worked together in Australia was a high point. Apparently, Zinkernagel had a fondness for singing soprano arias from Mozart operas in his basso voice. The two men's families were so engaged in the process that they have no photos of that intense time. Doherty and Zinkernagel's wives may be the real heroines of the 1996 prize, since they raised four preschool-aged children while holding jobs of their own. Doherty reflects that this may represent the kindness of women, and both couples have stayed together—not the usual case in modern life. I must confess that the stories of their personal lives were more engaging than the discussion of cell-mediated immunity that won them their Nobel.

Peter Doherty shares observations on a variety of topics that may not be too surprising to veteran scientists, but again his views may be very informative to nonscientific readers. He notes that, sadly, the Nobel Prize often permanently scars the recipient with unwanted demands that usually remove the privacy and protection needed for introspection and creativity. Apparently, most Nobel laureates never recover from the event, and many can no longer pursue their life's work at the former level of activity, if at all.

Unafraid to pull his punches, Doherty has some specific advice to government leaders. He clearly states that there is nothing to stop a United States president from appointing a top scientist to the Cabinet. Indeed, John F. Kennedy did so by naming the first US presidential science adviser, Jerome Wiesner, who subsequently became president of MIT. My own advice to current US First Lady Laura Bush is that Doherty's book would have been a valuable 60th birthday gift to the US commander in chief. Then he might realize that all Nobel laureates are not “Nobel Peace Prize winners,” as he is known to address the new awardees at their annual visit to the White House.

The press often seek Nobel laureates for comments, and Doherty plainly reveals the danger of such. Responding to an Australian journalist who asked about his opinion on opportunities to train abroad, Dougherty was frustrated to see his words misrepresented in print as “Nobel Prize winner tells young scientists to go to America.” It was even worse when his positive view of the American liberal arts education appeared under the headline “Nobel laureate suggests a society of ‘Know-It-Alls.’” At the same time, Doherty is critical of the often misinformed public for expecting that scientists will provide miraculous breakthroughs that absolve the public of responsibility.

The very worrisome issue of fraud in science is treated with considerable skill. In Doherty's opinion, this rare but extremely damaging event is often the product of a remarkable breakthrough that subsequently cannot be reproduced by the discoverer. Young scientists in particular need to be guided to avoid this fatal trap, and Doherty extols the need to know when to back away from a promising result.

Doherty's final chapter, entitled “How to Win a Nobel Prize,” is a rich source of pearls for the young scientist. It reflects the previous publication by Michael Bishop [4] as well as Santiago Ramon y Cajal's classic from 1898, *Advice to a Young Investigator*. I will stress just a few pieces of advice: (1) Learn to write clearly and concisely. The problem with much of science is that the scientists hurt their own efforts by being unintelligible. (2) Be generous and culturally aware. Acknowledge the achievements of others. Every young scientist needs frequent reminders that it is important to have as few enemies as possible. (3) Time is precious. Women in particular are vulnerable to “death by committees,” and their representation is sorely needed. (4) Avoid prestigious administrative roles. This is a major source of destruction, particularly to those from clinical backgrounds. Now a sometimes beleaguered vice chancellor, I can only underscore the importance of this point. (5) Live a long time. It may take 50 years for the Nobel recognition of a discovery!

Other topics could have been covered, such as the transience of Nobel celebrity. Quickly ask yourself, who won the Nobels two years ago? The book also does not discuss the irony where the conventional wisdom regarding a discovery or development does not coincide with the actual Nobel committee decisions. For example, the 1954 Nobel Prize for medicine was awarded to Enders, Wellers, and Robbins for their work on polio—not to Salk and Sabin. Similarly, the 1923 Nobel Prize for medicine went to Banting and MacLeod for the discovery of insulin—not to Banting and Best as commonly believed. So even winning a Nobel is not a guarantee of being remembered.

I look forward to reading future Nobel autobiographies, in particular, stories by the women Nobel laureates. Their views will be particularly useful, in light of the recent and dramatic emergence of women in certain scientific disciplines. And like many others, I hope that we will get the amazing inside story from two other Australians, Barry Marshall and Robin Warren, winners of the 2005 medicine prize, on how they discovered that peptic ulcer disease is caused by a lowly bacterium: it involves the unlikely combination of drinking a bacterial culture and surfing.

## 

**Figure:**
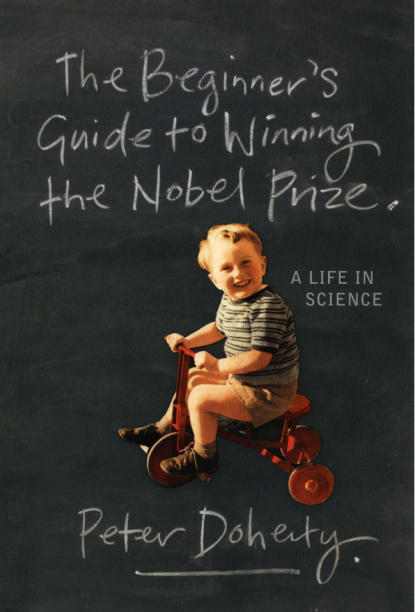
Doherty P (2006) The beginner's guide to winning the Nobel Prize. New York: Columbia University Press. 320 p. ISBN 0231138962. US$24.95
